# The Medical Parliamentary Committee

**Published:** 1919-02-08

**Authors:** 


### The Hospital

The Workers' Newspaper of Administrative Medicine and Institutional Life, Administration, National Insurance and Health.

No. 1705, Vol. LXV. SATURDAY, FEBRUARY 8, 1919. PRICE TWOPENCE

### THE MEDICAL PARLIAMENTARY COMMITTEE.

None know better than members of the medical profession that it is more easy to give good advice than to follow it. Exhortations and counsel may admittedly be of the highest wisdom, but their application is often sadly conditioned by circumstances. The guiding line may be clear as the sun at noonday, but the pursuit of it frequently needs some personal self-sacrifice and a willing mind. At the present moment the profession is surely realising the significance of these truths. Its future in many important respects is a highly uncertain quantity, and there is a risk that its status and the conditions of its existence may be fixed by outside authorities inadequately informed of its needs and desires. Little wonder, therefore, that all its counsellors urge upon the profession the necessity of union and the development of an organisation competent to speak with authority on its behalf. Every one will agree that this is excellent advice and that the neglect of it is likely to lead to a considerable disaster. That the advice is not being followed is notorious. It is true that there are many protests in its favour and that the general body of the profession is sincerely anxious that it may be accomplished. But among the leaders, or would-be leaders, there are not a few who, while they pay lip-service to the importance of unity, take action in an entirely opposite direction. What other judgment can be passed on those who, when the crisis is here and now, propose to establish new organisations for the collection and presentation of the resolved opinion of the profession? Their motives may be excellent, but their wisdom is a more doubtful quantity, and the effect of their proceedings is sure to be disastrous. Apart from other considerations the element of time is alone sufficient to render such enterprises useless, and the multiplication of sects emphasises and aggravates the opportunities for conflict and dissent. What fails the profession is not an organisation, but a lively interest and resolve to command and utilise opportunites already existent, and the true counsel to urge is not the creation of a number of "little Bethels," but the universal exercise of a franchise which is already within the reach of all. If, as is sometimes said, the largest existing medical organisaton does not truly represent the profession the position can be at once amended by the active intervention of those who claim to be the real voice of the constituency, for the machinery exists and is capable of rapid transfer to the elected majority. A devotion to the cause of union which expresses itself in the form of a new opportunity for diversion merits but scanty respect.

One of these newly manufactured organisations, if so it may be classed, illustrates very forcibly the dividing effect of the policy of multiplying independent committees and suchlike bodies. We refer to the so-called Medical Parliamentary Committee which, apparently with its own approval, is being presented to the public and the profession as an authoritative and representative professional organisation. The claim is a wholly unjustified one. Those who note such things will remember that a few months before the general election a meeting of the medical profession was called in a hall in the West-end of London. It was presided over by a much respected surgeon whose main contribution to the discussion was the advocacy of a proposition that the medical profession ought to enjoy special electoral privileges and thus be able to send direct representatives to the House of Commons. Whether his platform supporters agreed with him or not did not appear, for no further allusion was made to this topic, and all subsequent eloquence was devoted to the pleasing prospect of seeing a number of medical men attaining to Parliament by the aid of the ordinary constituencies. Naturally, this prospect appealed to professional patriotism, and there was a practically unanimous agreement that such an ambition ought to be supported. But how to support it was another question and here much difference of opinion found expression. Then came the platform proposition that a Committee be appointed to promote this aim, and a prepared and printed list of names was presented to the meeting. By this time a good deal of confusion had established itself, but ultimately the motions were declared carried, and later it was announced that the Committee as proposed had been elected. In addition, it was intimated that no effective action would be taken until the Committee had drafted its scheme and this had received the endorsement of another general meeting of the profession. Thus, at the best, this Committee rests on the votes of a relatively insignificant number of medical practitioners, and its sole reference was to prepare proposals for submission to a later discussion.

During the general election not much was heard of the Committee except through a trivial personal correspondence, from which it could be gathered that, while the Committee recommended certain candidates as suitable members of Parliament, it by no means recommended any constituencies to elect them. No second general meeting has been summoned and no report from the Committee has been published. Yet now we are told that a body having the origin and values here described is entitled to speak with representative authority and that to it we may look as to the nucleus of a medical parliament. That some of the members of this Committee are animated by a sincere desire to promote the unity and the welfare of the profession we have not the slightest doubt. But to claim the Committee as speaking the voice of the profession in some special and intimate fashion is little short of ludicrous. Large numbers of medical practitioners have never heard of its existence, and even its most assured supporters can only profess its right to draft a scheme to promote medical representation in Parliament. Any step beyond this restricted function is gratuitous and unwarranted, and so far from promoting union in the profession is likely to excite dissent and distrust. We are all for unity, but it must be pursued by open and proclaimed methods and must rest on a broad and liberal franchise.

